# Effects of predator lipids on dinoflagellate defence mechanisms - increased bioluminescence capacity

**DOI:** 10.1038/s41598-017-13293-4

**Published:** 2017-10-12

**Authors:** Jenny Lindström, Wiebke Grebner, Kristie Rigby, Erik Selander

**Affiliations:** 1 Department of Biological and Environmental Sciences, University of Gothenburg, Carl Skottsbergs gata 22B, SE 413 19 Göteborg, Sweden; 2 Department of Marine Sciences, University of Gothenburg, Carl Skottsbergs gata 22B, SE 413 19 Göteborg, Sweden

## Abstract

Short flashes of blue light (bioluminescence) from dinoflagellates can reduce copepod grazing of light-emitting cells. Other protective strategies against grazing are toxicity, reduced cell chain length and altered swimming patterns in different phytoplankton. Both toxicity and bioluminescence capacity in dinoflagellates decrease in copepod-free cultures, but toxin production can be restored in response to chemical alarm signals from copepods, copepodamides. Here we show that strains of the dinoflagellates *Lingulodinium polyedra* and *Alexandrium tamarense*, kept in culture for 14 and 9 years respectively, are capable of increasing their total bioluminescence capacity in response to copepodamides. The luminescence response to mechanical stimulation with air bubbles also increases significantly in *L. polyedra* after exposure to copepodamides. Effects on size, swimming speed and rate of change of direction in *L. polyedra* and *A. tamarense* were not detected, suggesting that post-encounter mechanisms such as bioluminescence and toxin production may constitute the dominating line of defence in these taxa. To our knowledge, this study provides the first evidence of changes in bioluminescence physiology as a response to chemical cues from natural enemies and emphasizes the importance of bioluminescence as an anti-grazing strategy.

## Introduction

Bioluminescence in dinoflagellates is considered to protect emitters from predation, and several studies report that luminescent cells benefit from reduced losses to grazing copepods^1,2^. Bioluminescence capacity of the dinoflagellates *Lingulodinium polyedra* and *Alexandrium tamarense* is kept under continuous physiological regulation. It is controlled, both in a diurnal rhythm with high bioluminescence capacity and excitability during the dark phase and almost none during the light phase^3–5^ and by photoinhibition of the bioluminescence by light exposure during the dark phase, when luminescence capacity is normally high^2,6–8^. A single *L. polyedra* cell has the capacity to flash only 1–4 times in succession and will not flash again until after recovery of the cellular mechanisms required for bioluminescence^9,10^. Luminescence is activated by mechanical stimulation of the dinoflagellate cell membrane and the mechanosensitivity is tuned such that luminescence is not activated by typical fluid shear levels found in the ocean interior but requires forces equivalent to an actual encounter with a grazing copepod^11–13^. Presence of the multiple physiological control systems described above suggest that bioluminescence comes with a high energetic cost and that the behaviour is well adapted to only be displayed at relevant stimuli.

In *L. polyedra* strains kept in culture for several years, the bioluminescence capacity is reported to decrease with 40-80% over time^14,15^. Decreased mechanosensitivity, with longer response latency time and demand for a stronger physical stimuli to stimulate luminescence in an *L. polyedra* strain older than seven years has also been described^15^. The loss of bioluminescence capacity is suggested to be linked to reduced photosynthesis in old autotrophic cell cultures^14^, as bioluminescence capacity of *L. polyedra* in each dark phase is directly correlated to both the amount of light and the time of light exposure in the previous light phase^7^. However, this cannot explain the observed loss in mechanosensitivity.

Production of toxic secondary metabolites also decreases over time in predator-free cultures of dinoflagellates and most strains of *L. polyedra* and *A. tamarense* are producing yessotoxins and saxitoxins respectively^16,17^. The toxic metabolites can however be re-induced by exposing the cells to grazer cues^18^. Cultured *Alexandrium minutum* became up to 20x more toxic in response to copepod grazers, indicating an active regulation of toxin production in agreement with the optimal defence theory^19,20^.

Further responses of microalgae to copepod grazers include reduced colony size in *Phaeocystis globosa*, cell chain length in the chain-forming diatom *Skeletonema marinoi* and the dinoflagellate *A. tamarense*, as well as reduced swimming speed in *A. tamarense* ^21–24^. Both reduced colony size and swimming speed decrease encounters with grazers in these species and are attributed to reception of chemical cues from the copepods, rather than their physical presence^23,24^. The compounds that induce toxin formation in *Alexandrium* cells have now been identified as a group of eight polar lipids, copepodamides^20^.

Here we hypothesize that loss of bioluminescence capacity in cultured cells is due to lack of stimulation from grazer cues and that the light capacity of *L. polyedra* and *A. tamarense* from grazer-free cultures should increase in response to copepodamides. Increased light producing capacity in response to copepodamides in *L. polyedra* and *A. tamarense* would also corroborate the defensive role of bioluminescence in dinoflagellates by fulfilling the predictions of the optimal defence theory, which states that defences should be regulated in response to the level of threat. The effects of copepodamides on *L. polyedra* and *A. tamarense* swimming behaviour and size is also investigated, as *A. tamarense* is known to change colony size and swimming behaviour in response to copepods to reduce encounter rates with grazers.

## Results

Effects of the copepodamide treatments on *L. polyedra* and *A. tamarense* luminescence, cell size, cell density, and swimming behaviour were measured after 48 hours of copepodamide exposure.

### Luminescence increase in response to copepodamides

**Figure 1:**
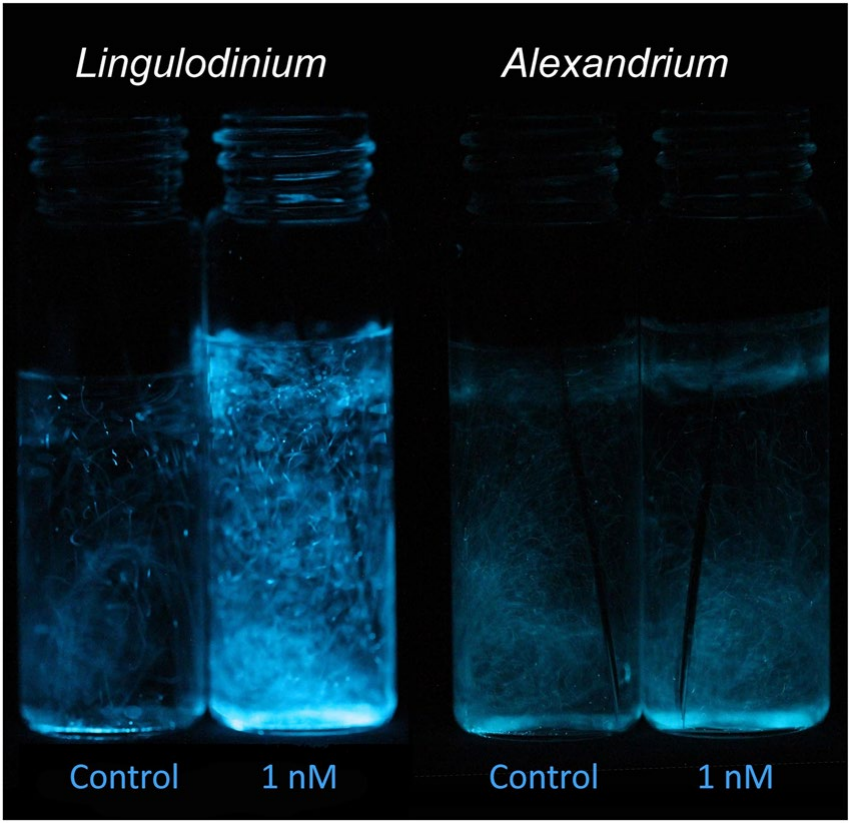
Image showing total luminescence from *L. polyedra* and *A. tamarense*. Cells are exposed to 1 nM copepodamides (right vial) or no copepodamides (control, left vial). Culture samples (5 ml) are simultaneously acidified by addition of acetic acid (1 M, 1 ml). *L. polyedra* cells produce visibly more light after pre exposure to copepodamides as compared to the control.

**Figure 2:**
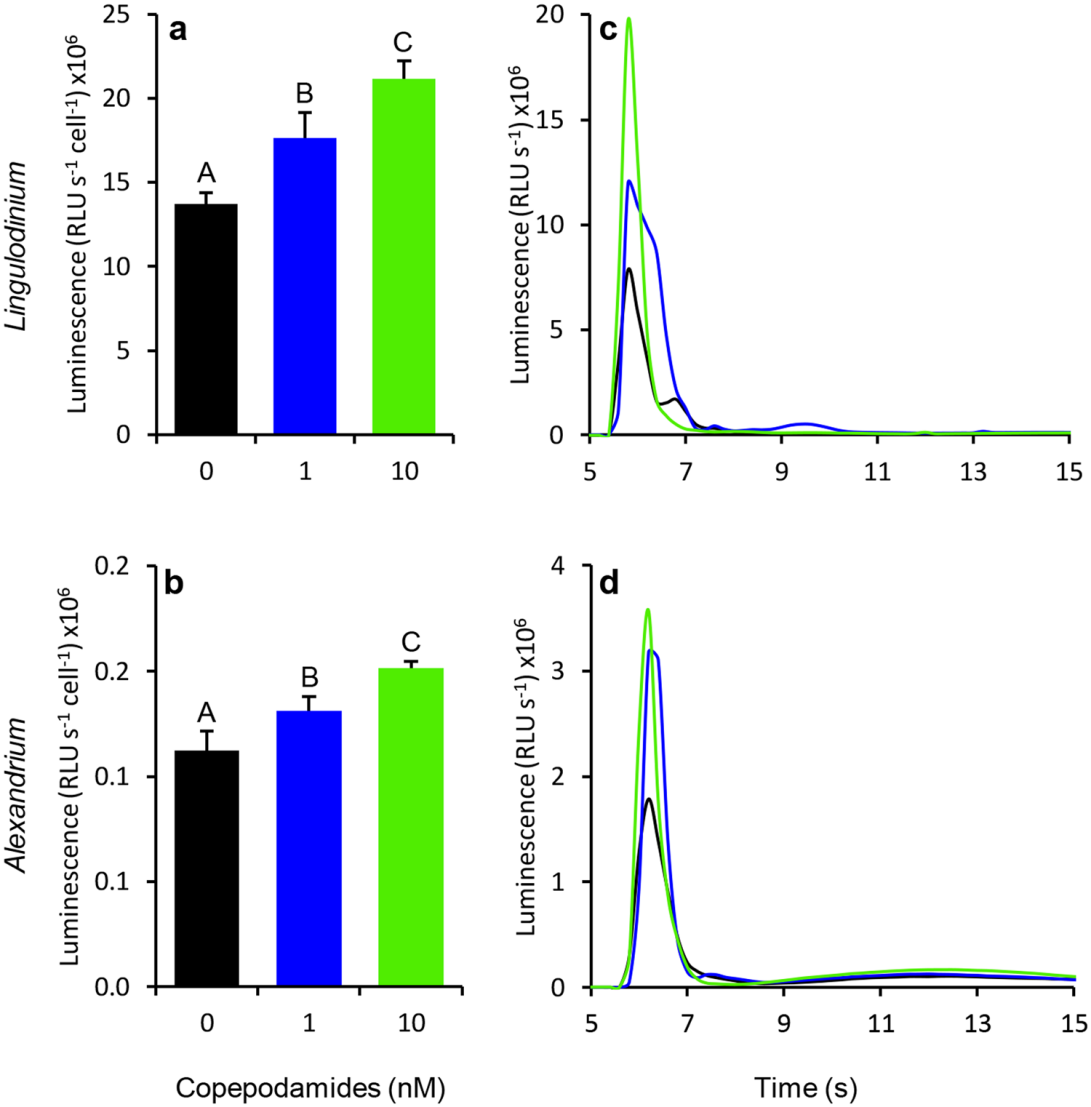
Total luminescence capacity of (**a**) *L. polyedra* and (**b**) *A. tamarense* exposed to 0, 1 and 10 nM copepodamides. (**a**) *L. polyedra* total luminescence capacity increased with increasing copepodamide concentration (p_ANOVA_ < 0.0001, p_SNK_ < 0.05, n = 4, bars denote mean ± s.e.m.). (**b**) *A. tamarense* luminescence capacity increased with increasing copepodamide concentration (p_ANOVA_ = 0.002, p_SNK_ < 0.05, n = 4, bars denote mean ± s.e.m.). Different letters above data bars indicate statistical difference between treatments. **(c–d**) Original recordings displaying the first 15 seconds of the luminescence response from a single sample of (**c**) *L. polyedra* (approx. 400 cells sample^−1^) and (**d**) *A. tamarense* (approx. 1000 cells sample^−1^) to acidification after treatment with 0 (black), 1 (blue) and 10 (green) nM copepodamides.

The copepodamide treatments induced a dose-dependent increase in total luminescence capacity, in both *L. polyedra* (Figs 1 and 2a) and *A. tamarense* (Fig. 2b). Total luminescence capacity was measured in Relative Light Units (RLU) at acidification of the culture samples. The *L. polyedra* total luminescence capacity increased by 28.8, and 54.4%, relative to the control, in response to 1 and 10 nM copepodamides respectively (p < 0.0001, Fig. 2a). The response in *A. tamarense* was less pronounced with 17 and 35% increase relative to the control (p = 0.002, Fig. 2b).

**Figure 3:**
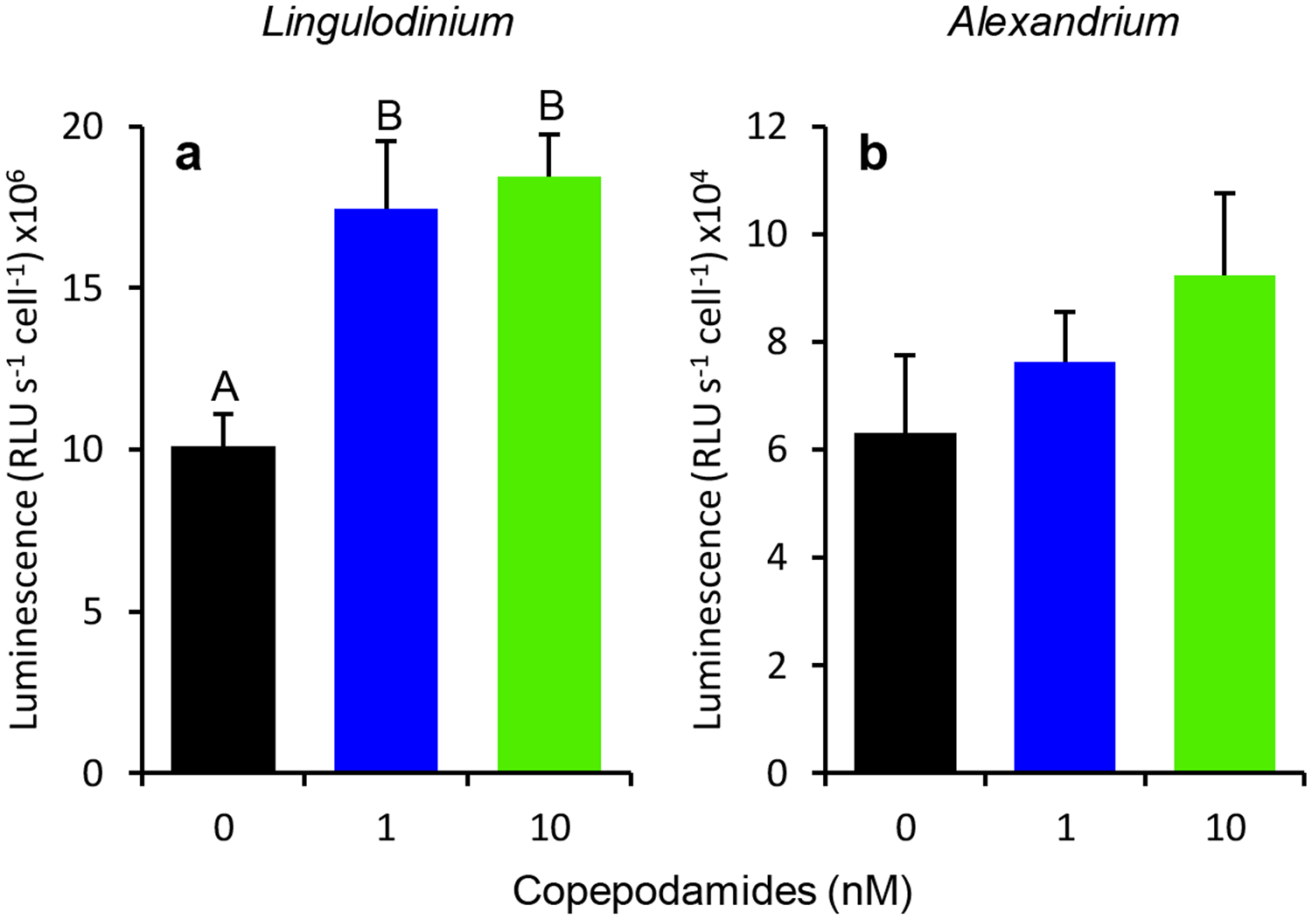
Luminescence response to mechanical stimulation by air bubbles for (**a**) *L. polyedra* and (**b**) *A. tamarense* exposed to 0, 1 and 10 nM copepodamides. (**a**) The response to air bubbles was significantly higher in *L. polyedra* pretreated with 1 and 10 nM copepodamides as compared to the control (p_ANOVA_ = 0.008, p_SNK_ < 0.05). (**b**) Response to air bubbles in 0, 1 and 10 nM copepodamide treated *A. tamarense* (p_ANOVA_ = 0.339). Different letters above data bars indicate statistical difference between treatments. n_all treatments_ = 4, bars denote mean ± s.e.m.

The luminescence response to mechanical stimulation (air bubbles) showed a similar pattern, but with larger effect size. Luminescence from mechanically stimulated *L. polyedra* cells increased by 73 and 83% relative to controls in the 1 and 10 nM copepodamide additions (p = 0.008, Fig. 3a). *A. tamarense* cells again showed a lower effect size, with 21 and 46% increase, however not significantly different from controls (p = 0.339, Fig. 3b).

### Cell size and density

**Figure 4:**
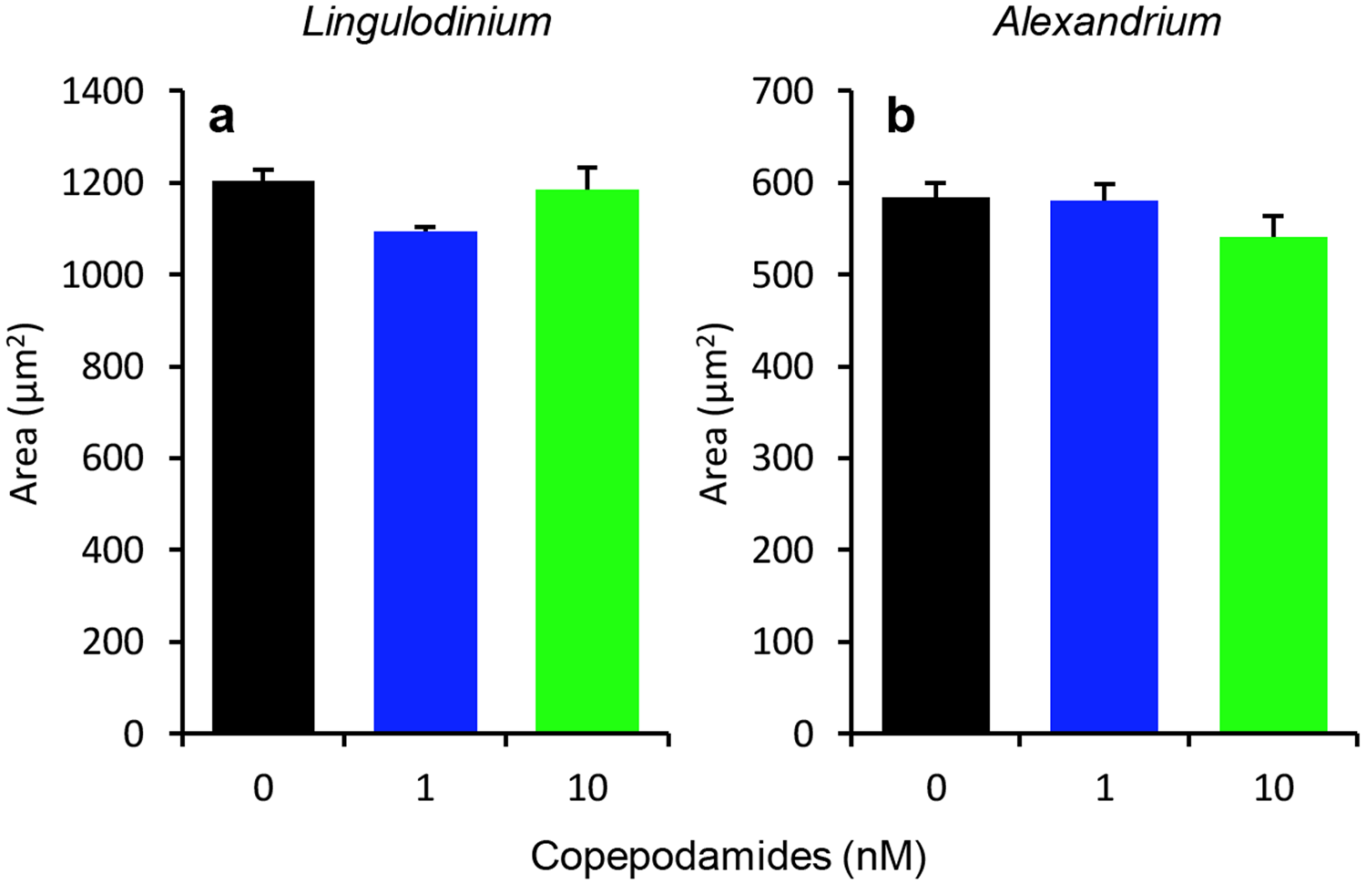
Effect of copepodamides on cell size in (**a**) *L. polyedra* and (**b**) *A. tamarense*. (**a**) Average cell area of *L. polyedra* after exposure to 0 (control), 1, or 10 nM copepodamides (p_ANOVA_ = 0.10). (**b**) Average cell area of *A. tamarense* after exposure to 0 (control), 1, or 10 nM copepodamides (p_ANOVA_ = 0.259). n_all treatments_ = 4, bars denote mean ± s.e.m.

In order to see if the treatment with copepodamides led to a reduced cell size in *A. tamarense* and *L. polyedra*, the cell area was measured. There were no significant effects of the treatments on the average cell area in *L. polyedra* (p = 0.1, Fig. 4a) or *A. tamarense* (p = 0.259, Fig. 4b). *A. tamarense* showed a slight decrease of 9% in the average area from 584 ± 16 µm^2^ (control) to 541 ± 23 µm^2^ (10 nM) (Fig. 4b). *L. polyedra* also showed a small decrease of 7.5% in the cell area from 1206 ± 24 µm^2^ (control) to 1095 ± 9 µm^2^ (1 nM) (Fig. 4a).

To rule out possible negative effects of the copepodamide treatments on cell growth and viability, the cell density was measured after 48 hours of copepodamide exposure. Cell density was not significantly affected by the copepodamide treatments as compared to the control, in *L. polyedra* (p = 0.15, control: 696 ± 17, 1 nM: 796 ± 41, and 10 nM: 739 ± 36 cells ml^−1^) and *A. tamarense* (p = 0.06, control: 2221 ± 68, 1 nM: 2117 ± 40, and 10 nM: 1970 ± 77 cells ml^−1^). The average cell density of *A. tamarense* for all treatments approximately doubled during the 48-hour incubation from 934 ± 71 cells ml^−1^ before start of the copepodamide incubation to 2103 ± 46 cells ml^−1^ after 48 hour of incubation. The larger *L. polyedra* did not grow during the incubation and had slightly lower density on average after the incubation (744 ± 21 cells ml^−1^) than before (934 ± 71 cells ml^−1^).

### Swimming behaviour in response to copepodamides

**Figure 5:**
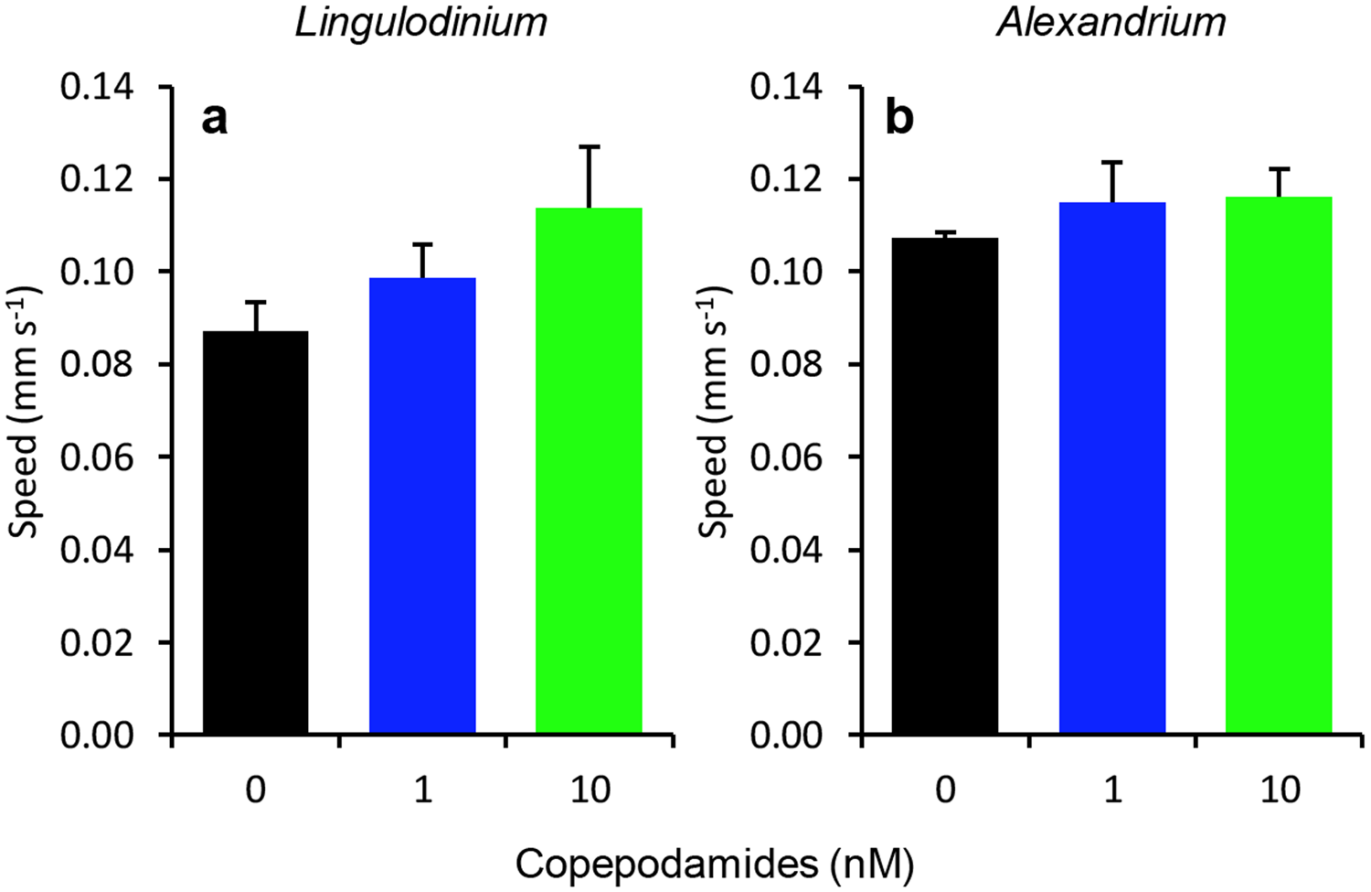
Effect of copepodamides on cell swimming speed in (**a**) *L. polyedra* and (**b**) *A. tamarense*. (**a**) Swimming speed of *L. polyedra* cells after exposure to 0 (control), 1, or 10 nM copepodamides, based on 534 individual tracks (p_ANOVA_ = 0.18). (**b**) Swimming speed of *A. tamarense* cells after exposure to 0 (control), 1, or 10 nM copepodamides, based on 695 individual tracks (p_ANOVA_ = 0.55). n_all treatments_ = 4, bars denote mean ± s.e.m.

**Figure 6:**
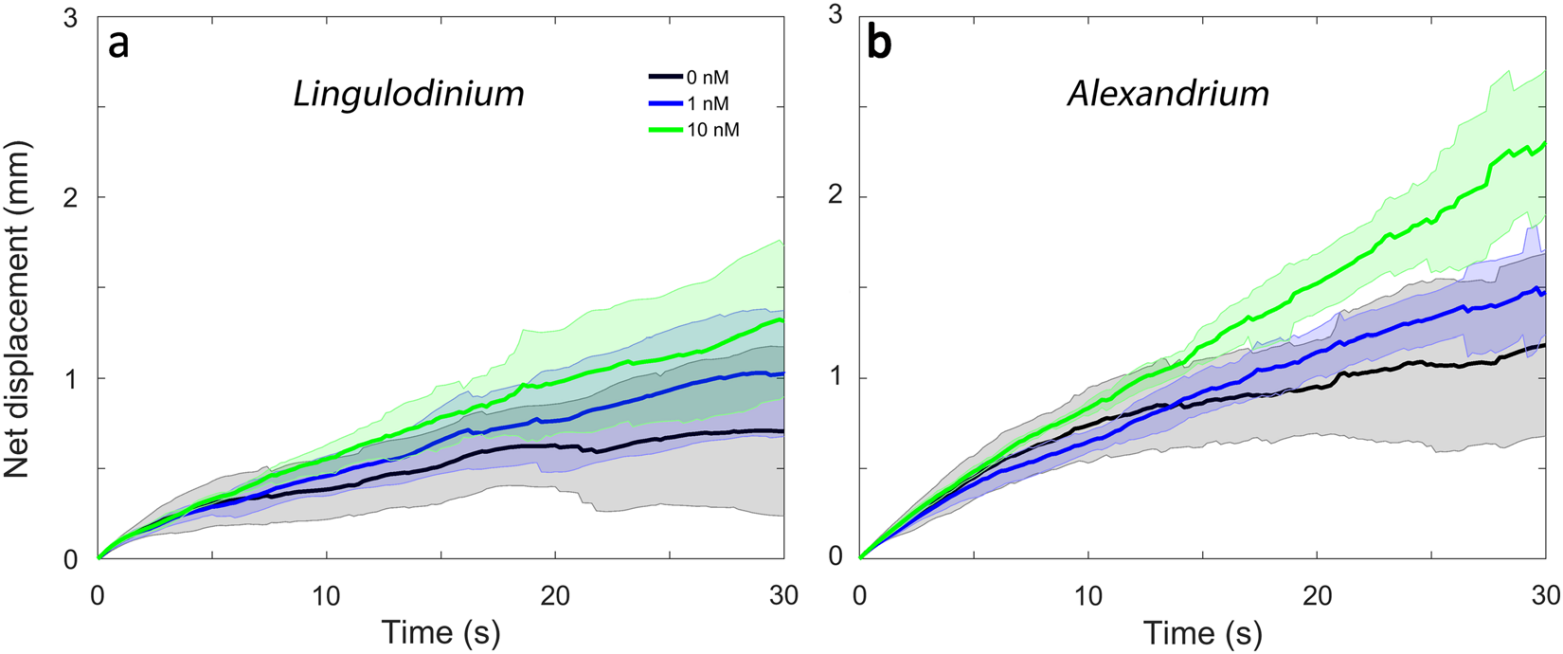
Effect of copepodamides on cell net displacement in (**a**) *L. polyedra* and (**b**) *A. tamarense*. (**a**) Net displacement of *L. polyedra* average trajectories obtained at 0, 1 and 10 nM copepodamides (p_ANOVA_ = 0.10). (**b**) Net displacement of *A. tamarense* average trajectories obtained at 0, 1 and 10 nM copepodamides (p_ANOVA_ = 0.13). Lines represents mean of four replicates with shaded standard deviation. Multiple trajectories were averaged from each replicate, in total 643 tracks for *L. polyedra* and 854 tracks for *A. tamarense*.

The average swimming speed did not change between treatments (p = 0.18 and 0.55 for *L. polyedra* and *A. tamarense* respectively, Fig. 5a,b). *L. polyedra* swam on average 100 ± 37 µm s^−1^ and *A. tamarense* 114 ± 37 µm s^−1^. Assuming isotropic swimming in all three dimensions this corresponds to a 3D swimming velocity of 140 and 122 µm s^−1^ for *L. polyedra* and *A. tamarense*, respectively. We monitored the net to gross displacement as more convoluted swimming trajectories reduce encounter rates with predators^25^. There was a trend towards less convoluted swimming patterns in copepodamide treated cultures, but the difference was not statistically significant (Fig. 6a,b, p = 0.10 and 0.13 for *L. polyedra* and *A. tamarense* respectively).

## Discussion

This study provides the first evidence of regulation of bioluminescence capacity in response to environmental cues in any species to our knowledge. It supports the defensive role of bioluminescence in dinoflagellates^1,26–29^, and suggests that defensive traits follow the predictions of the optimal defence theory. The optimal defence theory predicts that inducible or regulated defence levels should evolve when the level of risk is variable and the defence is costly^19^. Copepod density varies several fold over the seasons in northern Europe, the origin of the *A. tamarense* strain used in this study. The *L. polyedra* strain originates from southern California. In this area a 50% variation in copepod abundance between seasons has been reported^30,31^. Even if there is no exact measurement of the cost of bioluminescence, the associated structures and control mechanisms suggest that also the criteria for inducible defence are fulfilled. Inducible defence systems also require a reliable cue, a proxy for the level of threat that can be detected by the prey. Copepodamides seem to provide such a cue^20^.

In nature, copepodamide concentrations similar to the treatments used in this study, can be expected in patches with moderately high copepod densities. A single copepod of the species *Calanus finmarchicus* can produce up to 120 pmol copepodamides in a day and copepod densities of 10–100 individuals l^−1^ is common for smaller copepod species^20,31,32^. Thus the addition of 1 and 10 nM copepodamides per day is on par with natural supply of copepodamides.

The 10 nM copepodamide treatment increased the total bioluminescence capacity, assayed by acidification of cells, in both *L. polyedra* (54%) and *A. tamarense* (35%) as compared to the controls. The increase in *L. polyedra* corresponds roughly to the 40–80% loss of bioluminescence capacity reported in older *L. polyedra* isolates compared to more recent isolates^14,15^, suggesting that the *L. polyedra* cells have the physiological capability to restore full bioluminescence capacity in response to copepod cues. The reduced light emission in predator-free cultures is consequently likely to represent a less defended phenotype rather than a consequence of reduced photosynthetic capacity of aged cultures^14^. The reduced photosynthetic capacity may, however, represent reduced energy demand of the less defended phenotype.

The effect of 1 and 10 nM copepodamides on the luminescence response was 44 and 29% stronger in response to mechanical stimulation by air bubbling compared to the acid treatment in *L. polyedra*. In *A. tamarense* the trend was similar with 4 and 11% difference in effect size between the two types of stimuli, in the 1 and 10 nM treatments. A difference in effect size between the two stimuli (acidification and air bubbles) suggests that the mechanosensing signal transduction pathway is sensitized by copepodamides, allowing more efficient access to the bioluminescent chemicals upon mechanical stimulation of the cell. Therefore, bioluminescence could be enhanced by copepodamides acting on two different cellular pathways, the production of bioluminescent compounds and the mechanotransduction pathway.

The diurnal control and photoinhibition of bioluminescence provide two potential pathways by which the copepodamides can affect bioluminescence capacity and mechanosensitivity. In the diurnal rhythm the scintillons (vesicles containing the luminescent chemistry) as well as the bioluminescence chemistry, the luciferin, luciferase and luciferin binding protein are broken down at the end of the night phase, and resynthesized again at the end of the day phase and beginning of the following night phase^4,33,34^. It has also been speculated that the mechanosensitivity of the plasma membrane changes according to the same diurnal rhythm^35^, while photoinhibition is hypothesized to mainly act on the mechanical excitability of the cell^6,8^. The cellular mechanisms for diurnal control and photoinhibition of bioluminescence are currently largely unknown.

Upregulation of toxin production as a response to copepodamides seems to be dependent on cell division and takes place mainly after cell division^36^. In this study the cell count of *A. tamarense* doubled while the number of *L. polyedra* stayed the same over the copepodamide exposure time, indicating that each *L. polyedra* individual cell is capable of upregulating bioluminescence and mechanosensitivity as a response to copepodamides and that the mechanisms are independent of cell division. This is consistent with the diurnal regulation of bioluminescent capacity and photo inhibition mechanisms in *L. polyedra* ^4,5,37^ which are also faster than the cell cycle judging from laboratory growth rates.

Other phytoplankters are known to reduce colony size and swimming speed in response to grazer cues^21–24^. This was not the case here, since *L. polyedra* does not form chains and consequently could not change colony size. *A. tamarense* is known to form up to four-cell long chains but the *A. tamarense* culture used in this study had very few cells in two cell chains even in unexposed controls. Thus we cannot conclude if the copepodamides are the cueing compounds for chain length shortening in *A. tamarense* ^23^ or not. Moreover there was no significant effect on swimming speed or persistence of direction (as shown by the net to gross displacements in Fig. 6a,b) in either species as a response to copepodamides. Taken together, this suggests that post-encounter defensive traits such as bioluminescence and toxic secondary metabolites may be the primary means to counteract grazing in these dinoflagellates.

In conclusion the responsiveness of bioluminescent dinoflagellates to copepodamides in combination with lack of effect on cell size and swimming pattern emphasizes the importance of bioluminescence as an anti-grazing strategy in *L. polyedra* and *A. tamarense*. Dinoflagellates are large and slow growing. This makes them more vulnerable to top-down control from copepods compared to faster growing phytoplankton. Bioluminescence can offer dinoflagellates an advantage to other species in a situation with high abundance of copepods and thus have a potential of affecting the composition of the plankton community e.g. leading to blooms.

## Materials and Methods

### Organisms and culturing

*Lingulodinium polyedra* (strain CCAP 1121/5, isolated in 2003 from the American west coast, southern California) and *Alexandrium tamarense* (strain 3, isolated in 2008 from the Swedish west coast) were grown in F/2 medium^38^ supplemented with selenium (87 nM Na_2_ SeO_3_) in a growth chamber at 21 °C and about 50 µmol photons m^−2^ s^−1^ light. The cells were kept on a 12:12 h light:dark cycle. Cultures no more than three weeks post re-inoculation were used in the experiment.

Prior to the start of copepodamide exposure cultures from different flasks were pooled and gently mixed before dividing them in the treatment vials for incubation.

### Copepodamide treatments

*L. polyedra* and *A. tamarense* cultures were exposed to copepodamides extracted and purified from freeze dried *Calanus finmarchicus* as described in Selander *et al*. (2015)^20^. Glass tubes, with 80 ml volume and 22 mm inner diameter, were coated with the natural blend of copepodamides from *C. finmarchicus* dissolved in methanol (see Supplementary Table S1 and Graph S2 for composition of the copepodamide blend). The solvent was evaporated under a stream of N_2_ and the glass tubes filled with 30 ml culture leading to 1 nM (n = 4) or 10 nM (n = 4) final copepodamide concentration. The control tubes were coated with only methanol (n = 4). Growth of the cultures took place at the same conditions as described above. After 24 h the cultures were transferred into fresh tubes coated with the respective amount of copepodamides or methanol to assure constant exposure of the cells to the copepodamides. After further 24 h (in total 48 h) the cultures were used for the different experiments.

### Bioluminescence response

To assay the total luminescence capacity and luminescence response to mechanical stimulation in *L. polyedra* and *A. tamarense*, 0.5 and 1 ml subsamples of culture were gently pipetted into 5 ml plastic tubes. For mechanical stimulation four replicate samples were prepared. For the total luminescence capacity four true replicates with four additional technical replicates were prepared, resulting in 16 samples per treatment. All handling of cultures took place before the start of the dark phase, when bioluminescence is mechanically unexcitable. The samples were placed in the dark until start of experiment, 3 h into the dark phase when the cells are fully dark adapted and at the maximum light capacity^3,4^.

At the start of experiments culture samples were gently placed in a Berthold FB12 luminometer (Titrek-Berthold, Berthold Detection Systems GmbH, Pforzheim, Germany) with two neutral density filters (ND1.0, Edmund optics, York, United Kingdom) between the sample and the detector to avoid saturation of the detector. The order of samples was alternated (control, 1 nM treatment, 10 nM treatment, control, 1 nM treatment, 10 nM treatment and so on) to avoid bias due to possible temporal trends in luminescent capacity of the cells^3,4^.

To measure total luminescence capacity 0.5 ml acetic acid (1 M, Merck KGaA, Darmstadt, Germany) was added to a 0.5 ml culture sample 5 s after start of measurement through the injection head of the luminometer. Acidification resulting in intracellular pH < 6–7 activates the bioluminescence chemistry, independent of upstream physiological mechanisms and the light response is the total luminescence capacity of the cell^14,37,39^. The luminescence response was recorded for 2 min after which the luminescence was exhausted.

Air bubbling of culture samples was used to stimulate bioluminescence mechanically^3,40^. Prior to start of measurement a syringe needle (gauge 21) was gently inserted in a 1 ml culture sample through the injection head of the luminometer. Air was delivered through the needle to the sample via a 20 ml syringe and tubing at 8 ml min^−1^ speed using a syringe pump (Alladdin-1000, World Precision Instruments, Aston, Stevanage, UK). The luminescence response was recorded for 2 min.

Results are expressed in Relative Light Units s^−1^ cell^−1^ (RLU s^−1^ cell^−1^) as the accumulated light produced per cell during the time of measurement. Graphs show mean value of replicates ± standard error of the mean (s.e.m.) unless otherwise is stated. Prior to statistical analysis each sample data from the total luminescence capacity experiment was normalized to the average of the control and treatment data in one sample set (see above).

### Motion analysis

A well-mixed sample of 4 ml was gently pipetted into 6-well multi dishes, resulting in 4.2 mm liquid depth. The plate was left to acclimatize for ten minutes on a light table with cool light (Daylight, Spring TX, USA). Temperature during filming was 21.5 °C. Each replicate was filmed for 1 min at 5 fps using a monochrome camera (point grey BFLY-PGE-13E4M-CS, Point Grey, Richmond, Canada) fitted with a 55 mm micro Nikon macro lens operated at f 2.8. The focal plane was fixed at the same level above the bottom of the multi dish to avoid edge effects. One replicate from each treatment was included on each six well plate, and the order of video capture was reversed between each replicate to avoid systematic errors.

The swimming speed and rate of change of direction was quantified using the TrackMate plugin for Fiji ImageJ^41^. The image stack from each experiment was preprocessed with a macro and tracks exported to MATLAB (MathWorks, R2016a) (see Supplementary Methods S3 for macro scripts).

All tracks (1507 tracks) were included in the net displacement over time analysis. All tracks longer than 5 s were used to calculate swimming speed.  In total 1229 tracks with on average 51 tracks replicate^−1^ (min 16, max 107 tracks replicate^−1^). Average track duration was 23 s track^−1^.

### Cell counts

For determination of cell counts, 300 µl *L. polyedra* and 100 µl *A. tamarense* cell culture from each replicate was lugolized at T 0, 24 h and 48 h time points in a 48 or 96 well plate. Images were taken with the Point Grey BFLY-PGE-13E4M-CS monochrome camera and then analysed using a macro script (Supplementary Methods S3) in Fiji ImageJ (1.51 g) to automatically count the number of cells.

### Cell size

For size estimation 3 × 300 µl cell culture from each replicate were taken at time point 48 h and fixed with lugol’s solution in a 96 well plate. Pictures were taken under an inverted microscope (Axio vert.A1, Zeiss, Oberkochen, Germany) with the Dino-Eye microscope eyepiece camera (AnMo electronics corporation, New Taipei City, Taiwan). The size of at least 43 cells replicate^−1^ was analysed by measuring the cell area with the camera software (DinoCapture2.0 version1.5.11.A).

### Statistical analysis

Luminescence, swimming speed, cell density, and net to gross displacement results were tested with a one way ANOVA (IBM SPSS Statistics 24) with copepodamide concentration as a fixed factor followed by SNK post hoc test when applicable. Cell size was tested with a nested ANOVA (IBM SPSS Statistics 24). Statistical significance is based on p ≤ 0.05. Cochran’s test was used to test for homogenous variances prior to the ANOVA^42^.

### Data availability

All data generated or analysed during this study are included in this article and its Supplementary Information files. Raw data are available from the corresponding author on request.

## Electronic supplementary material

Supplementary information
