# Supplementary material for: Effects of predator lipids on dinoflagellate defence mechanisms - increased bioluminescence capacity
Source: Sci Rep. 2017 Oct 12;7:13104. doi: 10.1038/s41598-017-13293-4 (PMC5638803; doi:10.1038/s41598-017-13293-4)
Supplement: Supplementary file 1 — Supplementary information [file 41598_2017_13293_MOESM1_ESM.pdf]

Effects of predator lipids on dinoflagellate defence mechanisms - increased bioluminescence capacity

Lindström J., Grebner W., Rigby K. and *Selander E.*

Composition of copepodamide blend

The bioluminescent dinoflagellates *Lingulodinium polyedra* and *Alexandrium tamarense* were treated with a natural blend of 0, 1 or 10 nM copepodamides extracted from *Calanus finmarchicus*. The table (S1, below) show the contribution of each copepodamide to the total concentration in the treatments. The graphs (S2 A and B, below) are chromatograms from LC-MS analysis of the copepodamides in the blend. For the molecular structure of copepodamides, see Selander et al. 2015 [1].

Supplementary table S1 Composition of copepodamide blend extracted from *Calanus finmarchicus*.

Copepodamide	MW (Da)	Contribution to total copepodamide content (%)
A	760.5	11.3
B	734.5	1.9
C	708.5	1.2
D	758.6	68.5
E	732.5	8.0
F	706.5	4.1
658	658.5	0.8
686	686.5	4.0
G	450.3	0.0
H	448.3	0.1

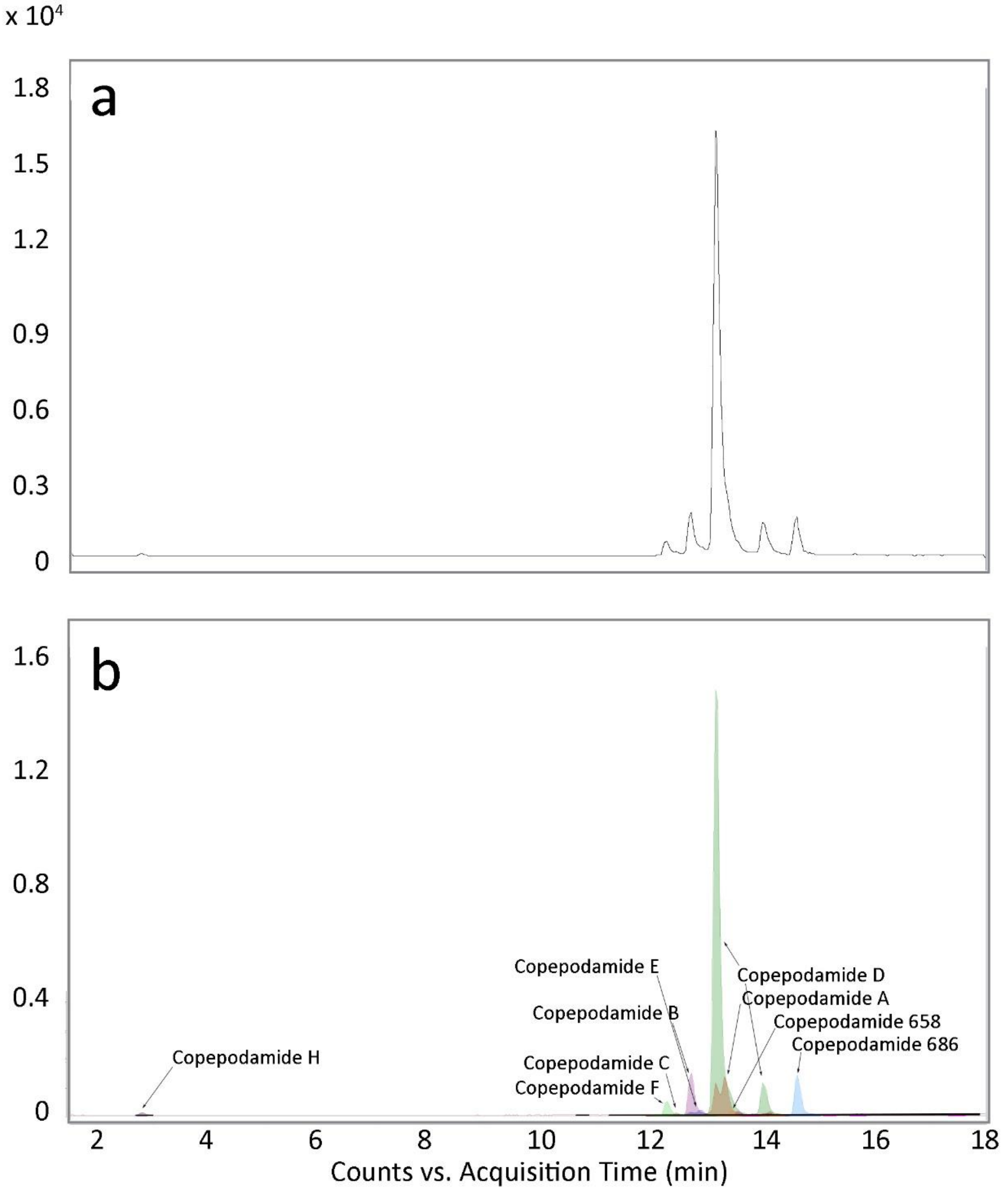

Supplementary graph S2 LC-MS chromatorgram analysis showing the total ion (A) counts from a MRM analysis of copepodamides extracted from *Calanus finmarchicus*. The integrated area of each individual copepodamide is shown in overlaid extracted chromatograms (B). The double peak labelled copepodamide A results from the isotope peak of copepodamide D with two ^{13}C resulting in the same mass as copepodamide A. The second, smaller peak of copepodamide D represents an un-identified isomer of copepodamide D. Copepodamide 658 and 686 represents two new copepodamides, with identical molecular scaffold (copepodamide H) as copepodamide D, E, and F, but with unidentified acyl group attached to C5 of the scaffold (see Selander et al 2015[1] for details).

Supplementary methods S3 ImageJ macro scripts for cell counting and motion analysis

Macros used in Fiji ImageJ (1.51g) to estimate cell density in *Alexandrium tamarense* and *Lingulodinium polyedra* cultures:

```
Macro for Alexandrium cell counts
dir1 = getDirectory("Choose Source Directory ");
dir2 = getDirectory("Choose Destination Directory ");
list = getFileList(dir1);
setBatchMode(true);
for (i=0; i<list.length; i++) {
showProgress(i+1, list.length);
open(dir1+list[i]);
stackli = getImageID();
run("Brightness/Contrast...");
run("Enhance Contrast", "saturated=0.35");
run("Apply LUT");
run("Close");
run("Subtract Background...", "rolling=10 light");
setOption("BlackBackground", false);
run("Make Binary");
run("Analyze Particles...", "size=5-50 pixel circularity=0.65-1.00
show=Outlines display exclude");
pic = getImageID();
imageCalculator("Average create", stackli,pic);
img_title = getTitle();
dest_filename = img_title+"original.tif";
fullpath = dir2 + dest_filename;
saveAs("tiff", fullpath);
selectWindow("Results");
saveAs("txt", fullpath);
run("Close");
run("Close");
run("Close");
}

Macro for Lingulodinium cell counts
dir1 = getDirectory("Choose Source Directory ");
dir2 = getDirectory("Choose Destination Directory ");
list = getFileList(dir1);
setBatchMode(true);
for (i=0; i<list.length; i++) {
showProgress(i+1, list.length);
open(dir1+list[i]);
stackli = getImageID();
run("Brightness/Contrast...");
run("Enhance Contrast", "saturated=0.35");
run("Apply LUT");

run("Subtract Background...", "rolling=10 light");

setOption("BlackBackground", false);
```

```
run("Make Binary");
run("Analyze Particles...", "size=15-70 pixel circularity=0.65-1.00
show=Outlines display exclude");
pic = getImageID();
imageCalculator("Average create", stackli,pic);
img_title = getTitle();
dest_filename = img_title+"original.tif";
fullpath = dir2 + dest_filename;
saveAs("tiff", fullpath);
selectWindow("Results");
saveAs("txt", fullpath);
run("Close");
run("Close");
run("Close");
}
```

Macro used in Fiji ImageJ (1.51g) to track cell movements in *A. tamarense* and *L. polyedra*[2]:

```
Macro for Tracking
//run("Brightness/Contrast...");
run("Enhance Contrast", "saturated=0.35");
run("Apply LUT", "stack");
run("Invert", "stack");
run("Subtract Background...", "rolling=15 stack");
run("Z Project...", "projection=[Average Intensity]");
imageCalculator("Subtract create stack", "Rep1","AVG_Rep1");
run("TrackMate");
*changed manually for each rep number
```

```
Settings (TrackMate)
Threshold= 1
check 'use median filter'
Frame to frame linking max distance= 0.25.
Track segment gap closing= 0.25 and Max frame gap= 2
Estimated blob size: L. polyedrum = 0.08, A. tamarense = 0.06
```

1 Selander, E. *et al.* Predator lipids induce paralytic shellfish toxins in bloom-forming algae. *Proc. Natl. Acad. Sci. USA* **112**, 6395-6400 (2015).

2 Tinevez, J. Y. *et al.* TrackMate: An open and extensible platform for single-particle tracking. *Methods* **115**, 80-90, doi:10.1016/j.ymeth.2016.09.016 (2017).
